# Response to “Field Trials Need Genetic Localization, Not Geographic Isolation”

**DOI:** 10.4269/ajtmh.25-0520b

**Published:** 2025-12-04

**Authors:** Gregory C. Lanzaro, Ana M. Kormos

**Affiliations:** University of California Malaria Initiative University of California Davis, California E-mail: gclanzaro@ucdavis.edu; University of California Malaria Initiative University of California Davis, California E-mail: amkormos@gmail.com

Dear Sir,

Dr. Esvelt proposes a so-called localized, threshold-dependent gene drive construct as a safer alternative to a low-threshold gene drive for malaria elimination in Africa.^1^ While such a construct may be suitable for modifying rodents on an affluent island in New England, it is neither sufficiently cost effective nor scalable to eliminate a disease, malaria, that kills half a million people each year in sub-Saharan Africa.

We argue that field trials of mosquitoes with low-threshold gene drives can be safely conducted on geographically isolated oceanic islands targeting populations that are demonstrably isolated genetically. We agree with global malaria experts that low-threshold drives are the only genetically modified (GM) mosquito approach that can meet the requirements for area-wide malaria elimination and, ultimately, continent-wide eradication. This strategy is cost-effective, and sustainable. We agree with Dr. Esvelt that field trials should have been conducted years ago, but these trials must be used to support an approach that will meet the stated goals and can only be achieved by groups that currently have products ready for testing.

Overall Dr. Esvelt’s letter exemplifies the fearmongering that often surrounds this science, employing recklessly alarmist language. He constructs a series of strawman scenarios to suggest that a field trial involving a low-threshold gene drive would be disastrous. His concern that a genetically engineered mosquito with a low-threshold gene drive (GEM_LTGD_) might escape from a field site and thereby jeopardize the entire gene drive approach is unfounded. Likewise, the prospect that some motivated anti-GM bioterrorists would transport engineered mosquitoes from a field trial site to locations outside seems antithetical to their beliefs and, even if bioterrorists chose to act in this fashion, they could just as easily transport a mosquito with a localized drive. So, it is unclear how this concern would justify supporting one approach over the other. Many of Dr. Esvelt’s concerns have been addressed by multiple research groups actively working in the field.^2^^–^^7^ Through detailed experimental analyses, including long-term assessments of the stability and efficacy of full-drive constructs maintained in laboratory conditions over several years, they have found no significant “unanticipated” risks. These important empirical studies, which Esvelt does not cite, should be given greater consideration as we move to solve serious problems in global health such as malaria.

A low-threshold gene drive can spread from very small initial releases and is highly invasive, while a threshold-dependent gene drive requires a minimum frequency in the population to propagate, thereby restricting its spread. To clarify, the gene drive we refer to is a low-threshold gene drive, the approach most widely recognized as having the potential to eliminate malaria from Africa.^8^^–^^10^ We advocate for a contained field trial of a GEM_LTGD_, the actual product that could be considered for broader deployment on the African mainland. Conducting trials with a localized construct would not be instructive. What is needed is a trial involving a mosquito that could realistically be used in a malaria elimination program in Africa.

A GEM_LTGD_ when released into a wild mosquito population, will increase in frequency to near fixation and subsequently spread beyond the release site via natural mosquito dispersal. The goal is to design a strategy for malaria elimination that is cost-effective, sustainable, and equitable. From the earliest discussions on the feasibility of genetically engineering mosquitoes for this purpose, it was recognized that a highly-efficient gene drive would be essential.^11^ Localized, or threshold-dependent gene drives are touted as being safe because they are assumed to have very limited spread and short lifespans. However, under certain ecological conditions they can indeed reach a frequency near 100% and can spread into neighboring populations, but their behavior in this regard is unpredictable.^12^

Low-threshold drives require the release of relatively small numbers of GEM_LTGD_ individuals at a limited number of sites. Unlike strategies based on localized constructs, which require prohibitively-costly and logistically-complex, continuous, repeated releases to sustain impact, low-threshold drives offer greater sustainability through autonomous spread. The construction and maintenance of large, costly GM mosquito rearing facilities, necessary for programs requiring repeated releases, are not required. Area-wide malaria elimination can be achieved through the natural dispersal of GEM_LTGD_ into surrounding regions as they move outward from an initial release site. These mosquitoes can reach rural areas where poor infrastructure makes direct releases difficult and expensive; therefore, the approach we advocate is equitable, reaching underserved communities where malaria imposes the greatest burden. GEM_LTGD_ will continue to persist and spread through the environment even in the face of civil unrest or pandemic conditions, such as COVID-19, that have disrupted other malaria control campaigns.

For clarity, when we refer to “geographic containment” in the context of genetically-engineered mosquitoes, we mean the containment of the mosquitoes themselves. Dr. Esvelt’s claim that “*geographic barriers cannot contain gene drives*” is simply incorrect. Barriers to gene flow are what shape the genetic structure of populations of essentially all sexually reproducing species. Such barriers even include limits to the movement of genes with major fitness advantages, such as gene drive elements.^13^

Population genomics studies of *Anopheles* mosquitoes from proposed field trial sites on isolated oceanic islands have demonstrated that these populations are genetically isolated, not only from mainland populations but even from neighboring islands.^14^^–^^16^

The notion that a few individuals carrying a low-threshold gene drive could “ultimately edit the bulk of an entire species” is highly implausible. A low-threshold gene drive is not a zero-threshold drive. These systems do require a minimum release ratio to spread effectively.^17^

Delaying the application of a technology that can save hundreds of thousands of lives and prevent millions of cases of clinical disease by focusing on unfounded, speculative, worse-case scenarios contributes to a cycle of stagnation that is unconscionable.
